# NEIGHBORHOOD PREDICTORS OF MENTAL HEALTH OF OLDER AMERICANS: EVIDENCE FROM A 5-YEAR LONGITUDINAL STUDY

**DOI:** 10.1093/geroni/igac059.1997

**Published:** 2022-12-20

**Authors:** Afolabi Orekoya, Henry Egbuchiem, Olawale Olanisa, Abimbola Arisoyin, Ujunwa Ebili, Adeleye Adaralegbe, Khuzeman Abbasi

**Affiliations:** Wellspring Medical Center, Riverdale, Georgia, United States; The Everest Foundation, Los Angeles, California, United States; IQVIA Biotech, Morrisville, North Carolina, United States; TCA health, Lagos, Lagos, Nigeria; Emel Specialists and Pediatric Center, Festac Town, Lagos, Nigeria; University of Connecticut, Storrs, Connecticut, United States, 7 Vanderbilt University Medical Center, Glen Burnie, Maryland, United States; Vanderbilt University Medical Center, Glen Burnie, Maryland, United States

## Abstract

With increasing dependence on other people in old age, environmental resources become an important asset for older adults to experience healthy aging. Data on the longitudinal relationship between neighborhood and mental health in late life is scanty. This study utilized hierarchical multiple regression model analysis to investigate whether and which neighborhood factors predicted depression and anxiety among older Americans followed up for over five years within the same neighborhood. Two waves of data containing a cohort of 1,731 older adults from the NSHAP project were used. Outcome measures were depression and anxiety. Predictors were four neighborhood factors: Social cohesion (NSC), social ties (NSC), neighborhood problems (NP), and perceived neighborhood danger (PND). We adjusted for demographic and physical health characteristics. The mean age of the respondents was 71.4 ± 6.5 years and were mostly females (55.5%). Lower NSC and a higher PND significantly predicted depression. However, the model only explained 2.8% of the variance in depression. In the covariate-adjusted model, none of the neighborhood factors predicted depression, but the model significantly improved to 32.5%. NP was the only significant predictor of anxiety in the final model and explained 27.8% of the variance in anxiety. Covariates, which are primary determinants of mental health disparity, have a much larger role to play. This study sheds some light on the complexity of the relationship between neighborhood and mental health in older adults. Future policy development and interventions should target improving both physical and social environments to enhancing the mental wellbeing of older adults.

